# Academy of Sciences

**Published:** 1854-02

**Authors:** 


Translated from the Gazette des Hopitaux.

*Academy of Sciences—Dec. 5th, 1853—Experiments on the poison of the Rattlesnake—the effects of the poison and the means of neutralizing its absorption.*

M. D. Brainard, Professor of Surgery in the Medical College, Chicago, Ill., communicated the following:

His experiments were made mostly on pigeons. The serpents belonged to the species known as the Crotalophrous trigeminus, a species of which the bite is considered less dangerous than that other Crotali, owing perhaps to their less size.

The author describes the symptoms which he has observed in animals bitten, and the results of the poison as seen in post-mortem examinations. Among the latter he notes the following:

1st. A change in the form of the red corpuscles of the blood, which in animals dead from the bite, approach more nearly the spherical form.

2d. An abundance of white corpuscles, grouped together and forming mammillated masses.

3d. When death has not been rapid, a very evidently liquid condition of the blood contained in the cavities of the heart.

In the Mammalia he has also observed that in cases in which death has not followed rapidly, there is a tendency to hœmorrhage from the mucous surfaces, and sometimes an appearance on the skin of petechial spots.

Among the symptoms observed during life, one of the most apparent, and which in pigeons is very easily observed, is constriction of the glottis. Tracheotomy, so usefully resorted to in poisoning by strychnine, is naturally indicated. The result of this operation is to retard but not to prevent death. Cups applied over the wound act in the same manner; they are more efficacious, but still insufficient. Nevertheless, the application of cups, by retarding the absorption of the poison, gives time to infiltrate the wound, and the surrounding tissues, with medical substances. Those which M. Brainard has tried, are the Lactate of Iron, and the Iodide of Potassium, both in a state of aqueous solution. They are introduced into the tissues by means of a little syringe, ingeniously contrived for the purpose. By the use of these two substances, employed in proper time and with the necessary precautions, he has in a large number of cases saved the lives of animals which without this treatment must necessarily have died. M. Brainard believes that the Iodide of Potassium is more certain in its action than the Lactate of Iron.

The article was referred to a committee, consisting of Messrs. Dumeril, Magendie, Flourens and Pelouze.

In another number of the *Gazette des Hopitaux*, we find the following: Society of Surgery, meeting of Dec. 6th, 1853, M. Denonvillieres President. M. Brainard, of the State of Illinois, candidate for the place of foreign corresponding member, read a memoir entitled "On the injection of Iodine into the tissues and cavities of the body for the cure of Spina Bifida, Chronic Hydrocephalus Œdema, Fibrinous Effusions, Œdematous Erysipelas, etc., etc."

Referred to a Committee composed of Messrs. Michon, Larrey and Monod. J.

